# Finding Influential Spreaders from Human Activity beyond Network Location

**DOI:** 10.1371/journal.pone.0136831

**Published:** 2015-08-31

**Authors:** Byungjoon Min, Fredrik Liljeros, Hernán A. Makse

**Affiliations:** 1 Levich Institute and Physics Department, City College of New York, New York, NY, United States of America; 2 Department of Sociology, Stockholm University, Stockholm, Sweden; 3 Institute for Futures Study, Stockholm, Sweden; Hangzhou Normal University, CHINA

## Abstract

Most centralities proposed for identifying influential spreaders on social networks to either spread a message or to stop an epidemic require the full topological information of the network on which spreading occurs. In practice, however, collecting all connections between agents in social networks can be hardly achieved. As a result, such metrics could be difficult to apply to real social networks. Consequently, a new approach for identifying influential people without the explicit network information is demanded in order to provide an efficient immunization or spreading strategy, in a practical sense. In this study, we seek a possible way for finding influential spreaders by using the social mechanisms of how social connections are formed in real networks. We find that a reliable immunization scheme can be achieved by asking people how they interact with each other. From these surveys we find that the probabilistic tendency to connect to a hub has the strongest predictive power for influential spreaders among tested social mechanisms. Our observation also suggests that people who connect different communities is more likely to be an influential spreader when a network has a strong modular structure. Our finding implies that not only the effect of network location but also the behavior of individuals is important to design optimal immunization or spreading schemes.

## Introduction

Identifying influential spreaders on social networks is crucial for its practical application in real-world epidemic and information spreading [1–7]. For instance, superspreaders need to be immunized with the highest priority in order to prevent the pandemic of an infectious disease [8–12]. They are also important for spreading of information in viral marketing [1, 2, 13, 14]. To this end, several predictors for influential spreaders based on the topological property of complex networks, including high degree [8, 15] *k*-core [10, 16, 17], betweenness centrality [18], PageRank [19], and many others [20] were tested for identifying influential spreaders [10–12].

Most studies, however, have overlooked how to apply to the real-world social systems which is a serious problem in a practical sense. Most proposed centralities except the degree, which is a local centrality, require the information of the whole network structure. But collecting this information is nearly impracticable in real social systems. Specifically, gathering information of relationships among individuals is inevitably incomplete and erroneous [21], since it cannot but be conducted for a partial sample of a whole population. Thus, searching for the influential spreaders with these centralities may not be plausible for real-world spreading phenomena. On the other hand, if whole connections in a network are accessible, direct measuring for the influence of a single node is possible by using model simulation on the network, which obviates the need for predicting influential spreaders. Consequently, in reality most predictors proposed for an influential spreader are either inapplicable or unnecessary.

Thus more realistic approaches based on the characteristics of people such as their behaviors are demanded for predicting influential people without the explicit information of network structure. The benefit of this method is an easy applicability for any kinds of social networks since one can obtain the probabilistic actions of agents by using a survey conducted from a population. Through a survey, we can estimate the probabilistic tendency of how connections are established for each individual, for instance, how probable is to make a new friend by introduction from another friend or the frequency to make new friends from different groups. We find that these human actions have a large influence on the subsequent spreading of information and therefore can be a reliable predictor of the node’s importance in a future epidemic or in a viral marketing campaign via targeting people identified by their probabilistic actions. In addition, such ranking obtained from surveys can also apply to the situations when the information for only some people is accessible.

The social mechanisms of link formation driving evolution of networks have been studied for a long time in order to explain and predict complex phenomena in society. A number of social mechanisms for connection establishments have been proposed in sociology [22, 23]. Thanks to the detailed records in online social networks that captures the action of every individual, it is now possible to quantify the frequency of occurrence of different types of mechanisms by directly observing social interactions [24]. Thus, recently, the frequencies of the social mechanisms for each person in a social network have been revealed from the full log of the activity in online social networks [24]. In this paper, we mainly focus on the effect of the social mechanisms of link formation on the epidemic influence but it is worth noting that temporal activity pattern can also affect the spreading dynamics and influence of people [25–28].

In this paper, we propose an approach to identify influential spreaders based on surveys on human behavior and social mechanisms that can be given to a population without the explicit information of networks. We decode the relation between people’s characteristics that can be obtained by a survey and their influence in spreading using the real-world datasets that contain the full information of network evolution. Through the analysis of large-scale evolving networks, we identify the effect of the microscopic link formation on macroscopic consequences in spreading. We find that the interaction to connecting a hub can facilitate epidemic spreading and thus can be a reliable predictor of people’s importance in future epidemics or viral marketing campaigns. We also find that people with high frequency to connect different communities are more likely to be an influential spreader for the case when a network is composed of strongly connected modules. This research has practical implications, since our finding can be adopted in reality requiring only the tendency of individuals’ behaviors. Furthermore, our results provide a guideline for behavior to the public, about how to behave at the beginning stage of epidemic.

## Materials and Methods

### Social mechanism

In this paper, the social mechanisms are referred to as the probabilistic tendency of each kind of interaction among people in a given social network. The social mechanisms do not directly mean the motivation behind the link creation because several different mechanisms may result in the same type of link formation and link formation may not be motivated by only the structure [24]. In addition, these mechanisms are not complementary one another, because a link can be established by multiple different mechanisms. For instance, a newly created link can appear following balance and exchange interactions at the same time.

We use four classes of social mechanisms underlying the link creation on a network based on the multitheoretical multilevel formalism [23] proposed in sociology: (1) Exchange interaction corresponds to a newly form reciprocal link meaning that a new link is established in the opposite direction of an existing link. (2) Balance interaction corresponds to a newly form tie that closes a triangle by a directed edge. (3) Collective action (or preferential attachment [29]) corresponds to a link that connects with well-connected people. To be specific, in this study, we measure the extent of the collective action of each link as a continuous value using the cumulative probability *F*(*k*_*i*_) of the excess degree distribution for a newly connected neighbor *i*. Here, *F*(*k*) = ∑_*k*_*j*_ < *k*_ *k*_*j*_ *q*(*k*_*j*_)/⟨*k*⟩ where *q*(*k*) is the degree distribution of a network and ⟨*k*⟩ represents the average degree of a network. (4) Structural hole interaction considers a newly created link that connects two different modules (communities). Community structure is identified by the local version of link community detection method [30] when a new link is established (Text B in S1 File).

These social mechanisms are assigned on an evolving network at the moment when the link is newly added following the analysis developed in [24]. While constructing the evolving network by adding the new connection in sequential order, we characterize each connection to the corresponding social mechanisms based on a network configuration at the given moment. After all links are formed, the frequencies of social mechanisms of the origin node, *i*, $a_{i}^{\text{exc}}$, $a_{i}^{\text{bal}}$, $a_{i}^{\text{ca}}$, and $a_{i}^{\text{sh}}$, where *i* is node index, are defined as the number of neighbors that were connected by the corresponding mechanism, respectively, exchange, balance, collective action, and structural hole (the sum of the extent for the collective action of all connected nodes) normalized by the total number of neighbors. To be specific, the frequency $a_{i}^{\alpha }$ of social mechanism *α* for node *i* is defined as $a_{i}^{\alpha }=\frac{n_{i}^{\alpha }}{k_{i}^{out}}$, where $n_{i}^{\alpha }$ is the number of links formed corresponding to *α* social mechanism and $k_{i}^{out}$ is the outdegree (the total number of new connections). Therefore, each variable ranges from zero to unity, and as *a*_*i*_ increases, the corresponding social interaction is more frequent.

We stress here that the extent of social mechanisms of link creation for each individual can be estimated in a real setting by the surveys given to the population. For instance, one first could ask people to list their contacts and then as a second stage ask questions about each contact [31]. For example, we could ask questions like, (exchange) did the person contact you first?, (balance) did the person have common friends with you when you contacted him/her?, (collective action) did the person have a lot of contacts when you contacted him/her?, (structural hole) did person belong to another group than you when you contacted him/her? Therefore, an estimate of *a*_*i*_ for each individual can be obtained from the surveys conducted for the population. On the contrary, most centralities including *k*-shell index [10], betweenness centrality [18], and PageRank [19] cannot be obtained by this way since they require global network information.

### Data sets

We examine two social networks of Internet dating services in Sweden [24, 32] and the forum of internet-mediated prostitution in Brazil [33]. These social networks represent potential pathways for epidemic spreading including sexually transmitted diseases. We use the data of the largest site *qx.se* for Nordic homosexual, bisexual, and transgender people in 2006 (QX). Actions of every individual in the community, including adding an individual to the favorite list and guestbook signing, were recorded for two months starting from Nov. 2005. We use adding favorite lists (QXF) and signing guestbook lists (QXG) among many activities. We also analyze pussokram *pussokram.com* dataset (POK) [32], which was a Swedish online dating site for friendship including flirting and non-romantic relations. The data contains a full log for 512 days starting from the day when the community was created in 2011. The POK network that we use in this study consists of message senders, receiver, and the timing of interactions in the community. Internet-mediated prostitution data (PRO) [33] comes from Brazilian online forum where sex-buyers evaluate prostitutes. We construct the PRO network by connecting sex-sellers with buyers. Since the PRO network is an undirected and bipartite graph, the exchange and balance interactions are not defined. In order to investigate the problem of identifying influential spreaders of information, we study the citation network in the posts of an online network service, *livejournal.com* (LJ), for information spreading on social networks [12]. One should note that the QX has already a large part of network (85 and 87% for the QXF and QXG, respectively) whereas the others starts at time *t* = 0. Table 1 gives the basic information of the datasets.

**Table 1:** Properties of real-world networks used in this study.

Network	Name	Number of nodes	⟨*k*⟩	Modularity [34]
QX.com favorite	QXF	80,407	13.07	0.4060
QX.com guestbook	QXG	59,854	7.10	0.3893
POK.com	POK	29,242	5.95	0.3992
Livejournal.com	LJ	315,936	3.56	0.6578
Prostitution	PRO	16,729	4.67	0.6294

⟨*k*⟩ is the average degree of the network. We use the fast-greedy community detection algorithm [34] for measuring modularity.

We can reconstruct the evolving connection of networks, following the precise timing when a tie has been established, in contrast to the observation of static snapshots of networks. In our datasets, we can observe every evolution of social networks with the time stamp of link creations. Following the time stamps, we create the evolving network of interacting people by adding the links in sequential order. To be specific, a link establishes between two people when they communicate each other for the first time. We stress here that the precise information of temporal evolution is essential to identify the social mechanisms for each link. The social mechanisms should be defined at the moment when a new link established [24]. Accumulated static networks do not keep the order of time that links established and thus are misleading about the social interactions. In this regard, our datasets containing the full log of network evolution allow us to define social mechanisms properly.

### Influential spreader

In order to assess the influence of people for epidemic spreading, we use the epidemic size *M*_*i*_ originating from a seed *i* in the susceptible-infected-recovered (SIR) model on the finally accumulated network [10]. The SIR model has been used to describe infectious disease for a long time [35]. At the same time, the SIR model is a plausible model of information spreading [10]. In the SIR model, each node can be in one of three states, susceptible, infected, or recovered (or removed). Initially, all nodes are in the susceptible state except for a single node in the infected state. At each time, the infected node spread a disease/information to a susceptible neighbor with infection probability *β*. At the steady state, we measure *M*_*i*_ as the fraction of finally infected nodes. We define a node with high *M*_*i*_ as highly influential.

We choose the infection probability *β* to be a value covering a small part of a network, *β* ≳ *β*_*c*_ where *β*_*c*_ is the epidemic threshold for percolation [35, 36]. When *β* ≫ *β*_*c*_, all seed produces similar epidemic size because spreading can cover almost all network regardless of where it originated from [37].

Here, we consider the problem of finding a single influential spreader. In real-world application, one may need to find the most influential sets of multiple spreaders that can spread disease and information to the largest part of the network [13]. In general, the problem of identifying the most influential multiple spreaders is far different from finding one single spreader because the nodes infected by the origins can be largely overlapped [10]. Therefore, it is worthwhile to mention that collecting top influential spreaders based on the single spreader scheme cannot be guaranteed to identify a set of multiple influential spreaders.

## Results

### Predictor for influential spreaders based on human activity

We recreate the entire network by adding all links in the order of time that they were established. In order to assess systematically the relation of the epidemic influence *M*_*i*_ with the social mechanisms as well as topological metrics, we use multilinear regression analysis [38] with the following model (Tables A-E in S1 File):

$$M_{i}=c_{0}+c_{1}a_{i}^{\text{exc}}+c_{2}a_{i}^{\text{bal}}+c_{3}a_{i}^{\text{ca}}+c_{4}a_{i}^{\text{sh}}+c_{5}k_{i}+c_{6}k_{i}^{\text{sh}}+c_{7}k_{i}^{2\text{sum}}+c_{8}k_{i}^{\text{sum}}+\epsilon .$$ (1)

Here, *k*_*i*_ is the degree of node *i*, $k_{i}^{\text{sh}}$ is the *k*-shell index [10] (Text A in S1 File). $k_{i}^{\text{sum}}$ is the sum of degree of the nearest neighbors $k_{i}^{\text{sum}}=\sum_{j\in V(i)}k_{j}$ where *V*(*i*) is the set of node *i*’s neighbors [12], $k_{i}^{2\text{sum}}$ is the sum of degrees of the next-nearest neighbors, $k_{i}^{2\text{sum}}=\sum_{j\in V_{2}(i)}k_{j}$ where *V*_2_(*i*) is the set of neighbors of node *i*’s neighbors [12], and *ϵ* is the error term. We assume that the epidemic influence is linearly dependent on the considered variables and the four social mechanisms are independent of each other. We introduce the topological metrics, since we are interested in how much information we captured using the social mechanisms tendencies {$a_{i}^{\text{exc}}$, $a_{i}^{\text{bal}}$, $a_{i}^{\text{ca}}$, $a_{i}^{\text{sh}}$} in comparison with the more common topological measurements, {*k*_*i*_, $k_{i}^{\text{sh}}$, $k_{i}^{\text{sum}}$, $k_{i}^{2\text{sum}}$}. In order to avoid biased observation due to the large fluctuation in the small degree region, we exclude the data of people with degree less than three from our analysis.

**Fig 1:**
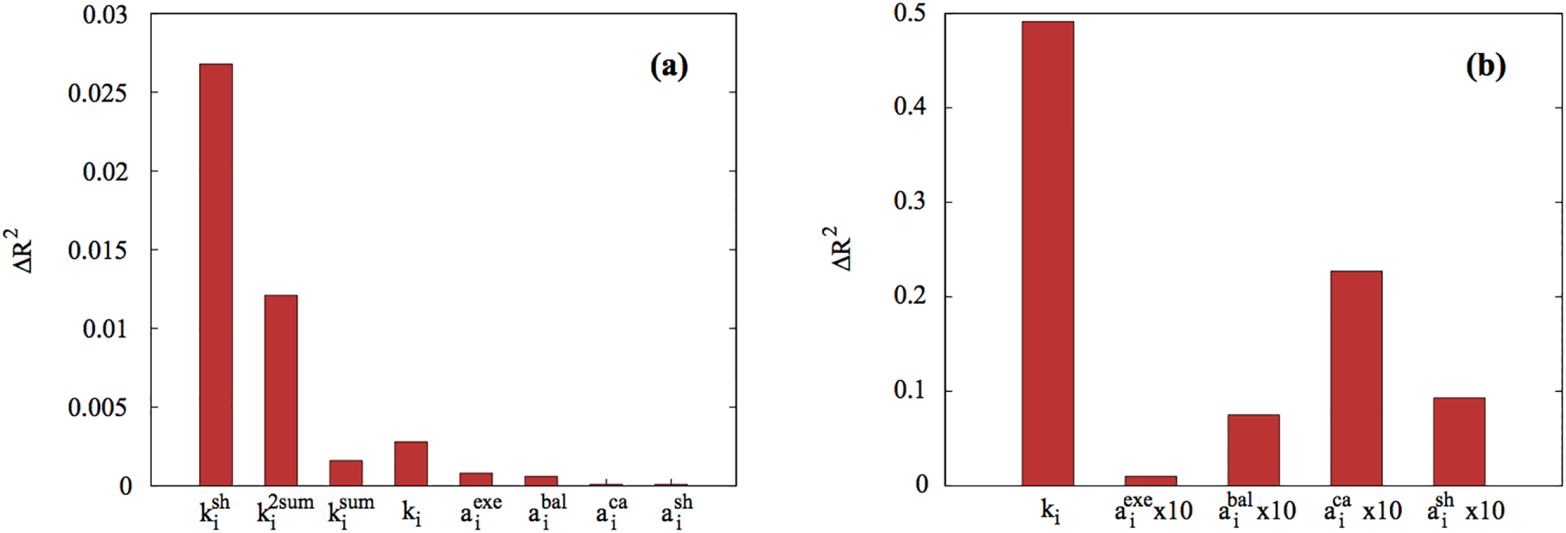
The difference Δ*R* ^2^(*x*) of the coefficient of determination when a variable *x* is excluded in regression analysis of (a) Eq (1) and (b) Eq (2) in QXF network. (a) *k*-shell index shows the largest drop of *R* ^2^, showing the strongest predictive power for influential spreaders. However, *k*-shell index is difficult to obtain since it requires the full topological information of the network. Although the degree and social mechanisms *a* ^*α*^ show smaller predictive power than *k*-shell index, they can be easily obtained from surveys and have much implication in a real setting. (b) The degree shows the largest difference among the degree and social mechanisms that can be obtained from surveys. Next, among the social mechanisms, collective action shows the largest drop of *R* ^2^. Thus, collective action is a more reliable predictor than the others from the human behavioral point of view.

The *k*-shell index and its local proxy *k* ^sum^ and *k* ^2sum^ have been regarded as an efficient topological predictor for influential spreaders [10, 12]. In agreement with these previous studies, we find that *k* ^sh^ can capture most of the fluctuation in the epidemic size for the datasets. To quantify the effect of each variable, we measure the difference Δ*R* ^2^(*x*) of the coefficient of determination when a variable *x* is excluded. In Fig 1, the difference Δ*R* ^2^(*k* ^ks^) of the coefficient of determination is the largest when *k* ^ks^ is excluded from Eq (1), which confirms the importance of *k* ^ks^. In addition, more than 82.3% of the fluctuations can be explained by solely the *k*-shell index for the QXF network (Table A in S1 File). For the QXG, POK, LJ, POK networks, we also find the similar trend as the QXF (Tables B-E in S1 File). However, being a global quantity, the *k*-shell index can be difficult to obtain as discussed above. Therefore, *k* ^sh^ has the limitation to apply for real social systems despite its strong correlation with the spreading influence. *k* ^sum^ or *k* ^2sum^ also captures a huge part of the variance in the data. While these are a local measurement, they still can be difficult to obtain because they require the exact number of friends of friends at the time when epidemic occurs [12].

The degree *k* is not behavioral but in contrary to *k* ^sh^, *k* ^2sum^, and *k* ^sum^, the degree *k* can be estimated by a survey to individuals by a simple question: how many friends do you have? Therefore, even if we cannot conceive the structure of network, for many cases, we can access the information of the degree together with the other social mechanisms, $a_{i}^{\alpha }$. Next we are interested in the case where the topological location such as *k*-shell cannot be obtained for the reasons explained above. Therefore, we regress the data of *M*_*i*_ with the variables which can be easily obtained by surveys using the following model, where *k* ^sh^, *k* ^sum^, and *k* ^2sum^ are excluded:

$$M_{i}=c_{0}+c_{1}a_{i}^{\text{exc}}+c_{2}a_{i}^{\text{bal}}+c_{3}a_{i}^{\text{ca}}+c_{4}a_{i}^{\text{sh}}+c_{5}k_{i}+\epsilon .$$ (2)

When we consider Eq (2), we can explain 63% of the variance for the QXF network (Table F in S1 File), demonstrating that with only surveys we can capture extremely high amount of the variance. The all variables in Eq (2) can be easily obtained from surveys, suggesting that we can rely on surveys for optimally immunization or viral marketing.

Using Eq (2), we find that the degree is the most reliable predictor for the influential spreaders among the degree and $a_{i}^{\alpha }$. When the degree is excluded from Eq (2), the coefficient of determination *R* ^2^ drops 0.49 from 0.62, showing the largest difference (Fig 1b). Since the degree represents the number of the transmission channels for a seed, the degree can play an important role in epidemic spreading on networks especially at the beginning stage of outbreak [10, 39]. When compared with the topological location of the people given by *k*-shell, we find that the degree alone can explain 58% of the variance, which compared to the value of *k*-shell (*R* ^2^ = 0.82), indicating that the degree is a worse predictor than *k*-shell in agreement with [10]. In a real setting, however, the local degree can have more implication than *k*-shell because it can be easily obtained from surveys.

Next, we are interested in what social mechanisms $a_{i}^{\alpha }$ are more important for spreading besides the local degree. This is not only important for optimal immunization and information spreading but also for education of the population to avoid certain behaviors that could spread diseases to huge population. In order to examine the effect of the social mechanisms clearly, we study the deviation of the epidemic size Δ*M*_*i*_ from the average epidemic size for people with the same degree by following

$$\Delta M_{i}=M_{i}-\frac{\sum_{j}\;\delta_{k_{i},k_{j}}M_{j}}{\sum_{j}\;\delta_{k_{i},k_{j}}},$$ (3)

where *δ*_*i*,*j*_ represents the Kronecker delta such that the function is 1 if the variables are equal and 0 otherwise. Δ*M*_*i*_ quantifies the impact of the social mechanisms after removing the effect induced by the degree, thus, more clearly identify the important social mechanism for spreading for people with the same degree.

**Fig 2:**
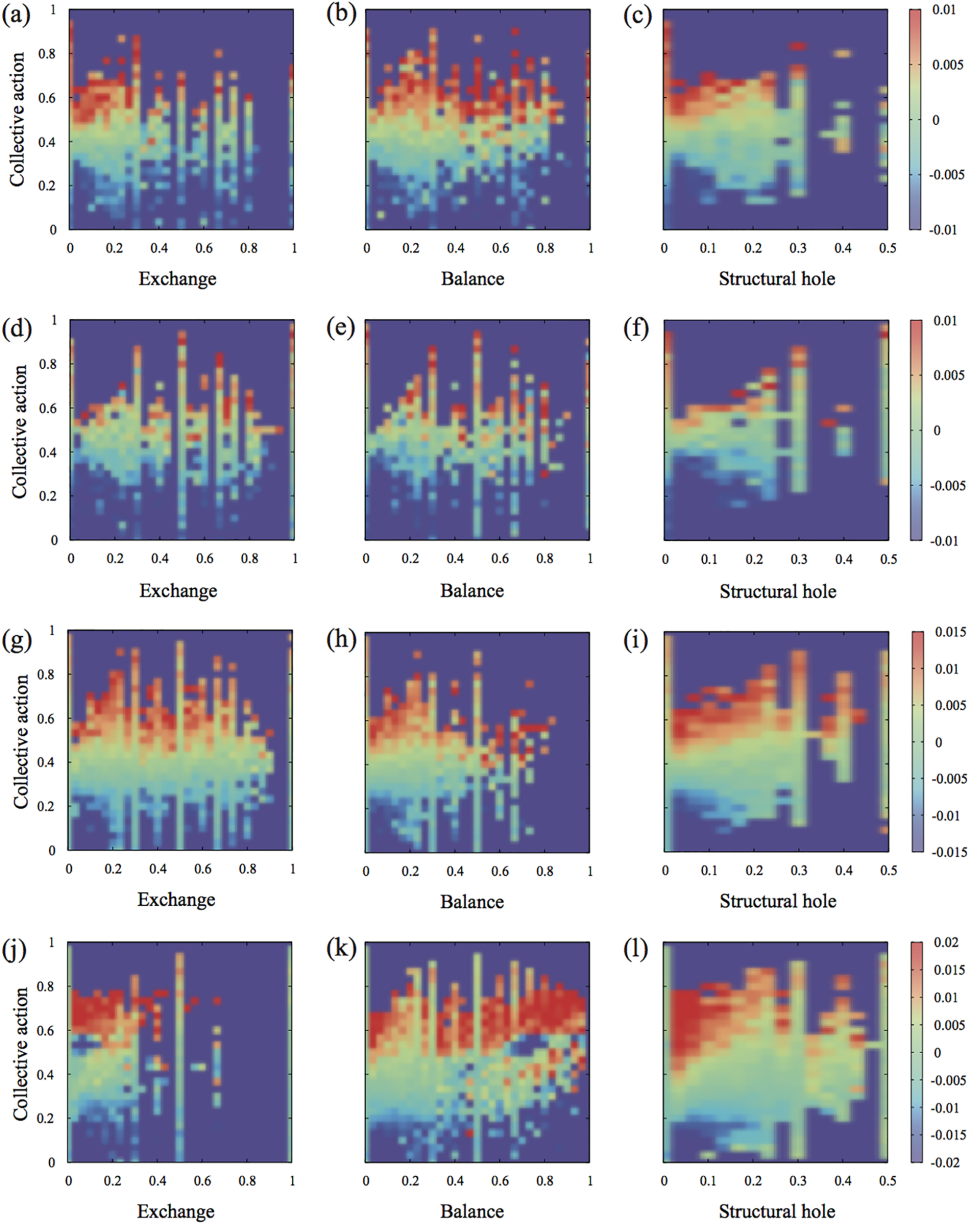
Collective action predicts influential spreaders more reliably than other social mechanisms. When spreading originates in people with (*a* ^*α*^, *a* ^ca^), the relative epidemic size *M*(*a* ^*α*^, *a* ^ca^) for the QXF with (a) *a* ^exc^, (b) *a* ^bal^, and (c) *a* ^sh^, (d-f) QXG, (g-i) POK, and (j-l) LJ networks. Collective action *a* ^ca^ predicts the epidemic influence more reliably than the other social interactions when we compare for people with the same degree.

To compare the influence of each social mechanisms in the spreading process, we study the average size Δ*M* infected in an epidemic originating at people *i* with a given ($a_{i}^{\text{exc}}$, $a_{i}^{\text{bal}}$, $a_{i}^{\text{ca}}$, $a_{i}^{\text{sh}}$). The average infected population over all the origins with the same pair of (*a* ^*α*^, *a* ^*β*^) is

$$\Delta M=\sum_{i\in W\;(a^{\alpha },a^{\beta })}\;\frac{\Delta M_{i}}{N\;(a^{\alpha },a^{\beta })},$$ (4)

where *W*(*a* ^*α*^, *a* ^*β*^) is the union of all nodes with (*a* ^*α*^, *a* ^*β*^) and *N*(*a* ^*α*^, *a* ^*β*^) is the number of nodes with (*a* ^*α*^, *a* ^*β*^). In Fig 2, we find that Δ*M* increases with increasing *a* ^ca^ regardless with the other social mechanisms for all tested networks. This clear pattern suggests that *a* ^ca^ predicts the epidemic influence more reliably than the other social interactions when we compare for people with the same degree.

The regression analysis of Eq (2) also supports the importance of the collective action. When we remove $a_{i}^{\text{ca}}$ from Eq (2), the difference Δ*R* ^2^ of the coefficient of determination is the largest, which confirms the importance of collective action. Since people with high collective action are more likely to have many next nearest neighbors, they have high chance to develop larger epidemic outbreaks. On the contrary, people with less collective action are likely to be located at the periphery of a network leading to a small impact in the spreading. Thus, the collective action is a reliable predictor from the human behavioral point of view when we factor out the popularity.

In contrary to the collective action, the exchange interaction plays a less role in spreading because it cannot give a new contact on networks. The balance interaction can establish a clique structure by making a triangle which can facilitate to form a core structure [40]. Thus, the balance interaction $a_{i}^{\text{bal}}$ can facilitate the formation of core structure and lead to positive correlation with the epidemic influence for most networks.

### Strength of weak ties and community structure

So far, we search the most influential spreaders based on social mechanisms, $a_{i}^{\alpha }$ which can be obtained by surveys. In sociology, a long-standing hypothesis for influential spreaders is the strength of weak ties [41]. According to the hypothesis, weak ties which bridge between two densely connected modules formed by strong ties play an important role especially in the job changing in labor market [41, 42], mobile communication networks [43], as well as brain [44]. While this hypothesis may seem counter-intuitive, for a perspective of information spreading, the weak ties are more likely to be a source of fresh information, so weak ties can have a stronger effect than strong ties.

In this section, we test the weak tie hypothesis by observing the evolution of link formation in a large scale real-world network. We define weak connection as a link bridging two different communities at the time when a new link is formed, called structural hole. If weak ties play an important role in spreading processes as the hypothesis of weak ties, people with high probability of structural hole interactions is more likely to have influence in spreading. In order to test the effect of weak ties (structural hole), we regress the data of *M*_*i*_ with the variables of social mechanisms $a_{i}^{\alpha }$ using the following model, where the network properties *k* ^sh^, *k* ^sum^, *k* ^2sum^, and *k* are excluded:

$$M_{i}=c_{0}+c_{1}a_{i}^{\text{exc}}+c_{2}a_{i}^{\text{bal}}+c_{3}a_{i}^{\text{ca}}+c_{4}a_{i}^{\text{sh}}+\epsilon .$$ (5)

The degree *k* is also excluded in order to focus on the effect of behavioral factors on spreading.

**Fig 3:**
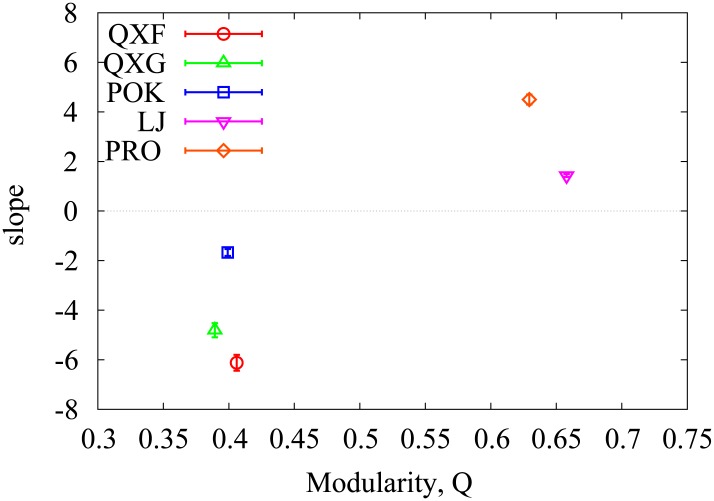
The effect of weak ties on spreading for different networks with diverse modularity. The panel shows the slope of the frequency of structural hole with respect to epidemic influence *M*_*i*_ in regression analysis as a function of modularity of a underlying network. For networks with highly modular structure such as LJ and PRO, the frequency of structural hole is positively correlated with *M*_*i*_.

From the regression analysis, we confirm that people with high frequency of structural hole interaction is more likely to be an influential spreaders on LJ and PRO networks as the weak tie hypothesis. In LJ and PRO networks, the frequency of structural hole *a* ^sh^ is positively related with the spreading influence *M*_*i*_ with extremely small p-value (Fig 3 and Tables I and J in S1 File). However, this pattern does not hold for all social networks that we tested. For QXF, QXG, and POK networks, $a_{i}^{\text{sh}}$ is negatively correlated with *M*_*i*_ in contrary to the weak tie hypothesis (Fig 3 and Tables F-H in S1 File). This result suggests that the weak tie hypothesis may not be generically valid for all social networks.

The validity of the weak tie hypothesis can rely on the underlying network where spreading occurs. People with high frequency of structural hole interactions potentially spreads different communities all together. Therefore, if an underlying network of spreading has clear module structure, the effect of weak ties is significant [45]. However, when community structure is less clear the role of weak ties in spreading can be weakened. In order to check this prediction, we compare the modularity of networks [46] and the effect of weak ties (Fig 3). When a network has strong community structure such as LJ and PRO whose modularity is 0.658 and 0.629, respectively, the frequency of structural hole is positively correlated with *M*_*i*_. Therefore, the structural hole mechanisms can enhance the epidemic influence for networks with strong modular structure as the weak tie hypothesis. However, the weak tie hypothesis is not valid for networks with less clear module structure. For instance, the QXF, QXG, and POK networks showing less modularity around 0.4, $a_{i}^{\text{sh}}$ play a minor role in spreading and negatively correlated with *M*_*i*_ (Fig 3 and Tables F-H in S1 File). If the modular structure is not significant, the weak ties are not clearly defined, leading to decrease of the effect of weak ties. Thus, the weak tie hypothesis is expected to be valid for strong module structure not universally for all social networks. In conclusion, people who connect different communities can be suspected as an influential people when an underlying network is composed of strong modular structure.

## Discussion

So far, most studies of spreading on complex networks have assumed that a network structure is known. This means that full information on any people on who is connected with whom is required, which may not be obtained in real settings. In agreement with the previous studies, we find that when the information of global structure of social networks is available, it is beneficial for identifying influential spreaders in an epidemic model capturing up to 90% of the variance with simple variables with the *k*-shell [10, 12]. In reality, however, it is difficult to gather the complete sets of interactions among people. Therefore, all the previous method for the influential spreaders based on the network topology could be impractical. Searching for influential spreaders without the information of a network is essential in order to prevent the global pandemic and minimize the cost for immunization.

Thus, we proposed a possible strategy for identifying the influential spreaders by using characteristics of people’s behavior underlying the evolution of social networks. Our finding provides several pragmatic lessons for the efficient immunization strategy as well efficient information spreading campaigns. First, in the absence of *k*-shell, the degree is the first local quantity that can be used to predict the influential spreaders. From the behavioral variables quantifying the social mechanisms $a_{i}^{\alpha }$, collective action gives a complementary information to the degree, so it is suitable for a strong indicator for influential spreaders when comparing among people with the same degree. Also, a person with a high tendency to connect two different groups via weak ties can also be suspected as an influential spreader when the network has a strong modular structure. Our analysis provides not only an applicable identifying scheme of influential spreaders based on surveys but also a guideline for activity to the public, about how to behave when epidemic occurs. For instance, during the beginning stage of epidemic, one need to avoid meeting popular people or people belonging to a different group that could spread diseases to huge population.

## Supporting Information

S1 File This file contains Tables A to J for multilinear regression analysis and supplementary methods for *k*-shell index and identifying structural hole in the link community. Table A, Multilinear regression for the QXF networks with Eq (1). Table B, Multilinear regression for the QXG networks with Eq (1). Table C, Multilinear regression for the POK networks with Eq (1). Table D, Multilinear regression for the LJ networks with Eq (1). Table E, Multilinear regression for the PRO networks with Eq (1). Table F, Multilinear regression for the QXF networks with Eqs (2) and (5). Table G, Multilinear regression for the QXG networks with Eqs (2) and (5). Table H, Multilinear regression for the POK networks with Eqs (2) and (5). Table I, Multilinear regression for the LJ networks with Eqs (2) and (5). Table J, Multilinear regression for the PRO networks with Eqs (2) and (5). Text A, *k*-shell index. Text B, Identifying structural hole in the link community.
(PDF)

## References

[1] Domingos P, Richardson M. Mining the network value of customers. In: Proceedings of the seventh ACM SIGKDD international conference on Knowledge discovery and data mining. ACM; 2001. p. 57–66.

[2] Richardson M, Domingos P. Mining knowledge-sharing sites for viral marketing. In: Proceedings of the eighth ACM SIGKDD international conference on Knowledge discovery and data mining. ACM; 2002. p. 61–70.

[3] Cohen R, Havlin S, Ben-Avraham D. Efficient immunization strategies for computer networks and populations. Physical Review Letters. 2003;91(24):247901 10.1103/PhysRevLett.91.247901 14683159

[4] Holme P. Efficient local strategies for vaccination and network attack. EPL (Europhysics Letters). 2004;68(6):908 10.1209/epl/i2004-10286-2

[5] Chen Y, Paul G, Havlin S, Liljeros F, Stanley HE. Finding a better immunization strategy. Physical Review Letters. 2008;101(5):058701 10.1103/PhysRevLett.101.058701 18764435

[6] Chen D, Lü L, Shang MS, Zhang YC, Zhou T. Identifying influential nodes in complex networks. Physica A: Statistical Mechanics and its Applications. 2012;391(4):1777–1787. 10.1016/j.physa.2011.09.017

[7] Chen DB, Gao H, Lü L, Zhou T. Identifying influential nodes in large-scale directed networks: the role of clustering. PLoS ONE. 2013;8(10):e77455 10.1371/journal.pone.0077455 24204833 PMC3814409

[8] Albert R, Jeong H, Barabási AL. Error and attack tolerance of complex networks. Nature. 2000;406(6794):378–382. 10.1038/35019019 10935628

[9] Holme P, Kim BJ, Yoon CN, Han SK. Attack vulnerability of complex networks. Physical Review E. 2002;65(5):056109 10.1103/PhysRevE.65.056109 12059649

[10] Kitsak M, Gallos LK, Havlin S, Liljeros F, Muchnik L, Stanley HE, et al Identification of influential spreaders in complex networks. Nature Physics. 2010;6(11):888–893. 10.1038/nphys1746

[11] Castellano C, Pastor-Satorras R. Competing activation mechanisms in epidemics on networks. Scientific Reports. 2012;2 10.1038/srep00371 22523634 PMC3330633

[12] Pei S, Muchnik L, Andrade JS Jr, Zheng Z, Makse HA. Searching for superspreaders of information in real-world social media. Scientific Reports. 2014;4 10.1038/srep05547 PMC4080224 24989148

[13] Kempe D, Kleinberg J, Tardos É. Maximizing the spread of influence through a social network. In: Proceedings of the ninth ACM SIGKDD international conference on Knowledge discovery and data mining. ACM; 2003. p. 137–146.

[14] Kempe D, Kleinberg J, Tardos É. Influential nodes in a diffusion model for social networks In: Automata, languages and programming. Springer; 2005 p. 1127–1138.

[15] Cohen R, Erez K, Ben-Avraham D, Havlin S. Breakdown of the Internet under intentional attack. Physical Review Letters. 2001;86(16):3682 10.1103/PhysRevLett.86.3682 11328053

[16] Dorogovtsev SN, Goltsev AV, Mendes JFF. K-core organization of complex networks. Physical Review Letters. 2006;96(4):040601 10.1103/PhysRevLett.96.040601 16486798

[17] Carmi S, Havlin S, Kirkpatrick S, Shavitt Y, Shir E. A model of Internet topology using k-shell decomposition. Proceedings of the National Academy of Sciences. 2007;104(27):11150–11154. 10.1073/pnas.0701175104 PMC1896135 17586683

[18] Freeman LC. Centrality in social networks conceptual clarification. Social Networks. 1979;1(3):215–239. 10.1016/0378-8733(78)90021-7

[19] Brin S, Page L. Reprint of: The anatomy of a large-scale hypertextual web search engine. Computer Networks. 2012;56(18):3825–3833. 10.1016/j.comnet.2012.10.007

[20] Pei S, Makse HA. Spreading dynamics in complex networks. Journal of Statistical Mechanics: Theory and Experiment. 2013;2013(12):P12002 10.1088/1742-5468/2013/12/P12002

[21] Lee SH, Kim PJ, Jeong H. Statistical properties of sampled networks. Physical Review E. 2006;73(1):016102 10.1103/PhysRevE.73.016102 16486211

[22] Monge PR, Contractor NS. Theories of communication networks. Oxford University Press; 2003.

[23] Contractor NS, Wasserman S, Faust K. Testing multitheoretical, multilevel hypotheses about organizational networks: An analytic framework and empirical example. Academy of Management Review. 2006;31(3):681–703. 10.5465/AMR.2006.21318925

[24] Gallos LK, Rybski D, Liljeros F, Havlin S, Makse HA. How people interact in evolving online affiliation networks. Physical Review X. 2012;2(3):031014 10.1103/PhysRevX.2.031014

[25] Vazquez A, Racz B, Lukacs A, Barabasi AL. Impact of non-Poissonian activity patterns on spreading processes. Physical Review Letters. 2007;98(15):158702 10.1103/PhysRevLett.98.158702 17501392

[26] Iribarren JL, Moro E. Impact of human activity patterns on the dynamics of information diffusion. Physical Review Letters. 2009;103(3):038702 10.1103/PhysRevLett.103.038702 19659326

[27] Min B, Goh KI, Vazquez A. Spreading dynamics following bursty human activity patterns. Physical Review E. 2011;83(3):036102 10.1103/PhysRevE.83.036102 21517553

[28] Muchnik L, Pei S, Parra LC, Reis SD, Andrade JS Jr, Havlin S, et al Origins of power-law degree distribution in the heterogeneity of human activity in social networks. Scientific Reports. 2013;3 10.1038/srep01783 23648793 PMC3646275

[29] Barabási AL, Albert R. Emergence of scaling in random networks. Science. 1999;286(5439):509–512. 10.1126/science.286.5439.509 10521342

[30] Ahn YY, Bagrow JP, Lehmann S. Link communities reveal multiscale complexity in networks. Nature. 2010;466(7307):761–764. 10.1038/nature09182 20562860

[31] Fridlund V, Stenqvist K, Nordvik MK. Condom use: The discrepancy between practice and behavioral expectations. Scandinavian Journal of Public Health. 2014;42(8):759–765. 10.1177/1403494814550518 25260638

[32] Holme P, Edling CR, Liljeros F. Structure and time evolution of an Internet dating community. Social Networks. 2004;26(2):155–174. 10.1016/j.socnet.2004.01.007

[33] Rocha LE, Liljeros F, Holme P. Information dynamics shape the sexual networks of Internet-mediated prostitution. Proceedings of the National Academy of Sciences. 2010;107(13):5706–5711. 10.1073/pnas.0914080107 PMC2851932 20231480

[34] Clauset A, Newman ME, Moore C. Finding community structure in very large networks. Physical Review E. 2004;70(6):066111 10.1103/PhysRevE.70.066111 15697438

[35] Anderson RM, May RM, Anderson B. Infectious diseases of humans: dynamics and control. vol. 28 Wiley Online Library; 1992.

[36] Newman ME. Spread of epidemic disease on networks. Physical Review E. 2002;66(1):016128 10.1103/PhysRevE.66.016128 12241447

[37] Pei S, Muchnik L, Tang S, Zheng Z, Makse HA. Exploring the complex pattern of information spreading in online blog communities. PLoS ONE. 2015;10(5):e0126894 10.1371/journal.pone.0126894 25985081 PMC4436014

[38] Gujarati DN. Basic econometrics. Tata McGraw-Hill Education; 2012.

[39] Pastor-Satorras R, Vespignani A. Epidemic spreading in scale-free networks. Physical Review Letters. 2001;86(14):3200 10.1103/PhysRevLett.86.3200 11290142

[40] Serrano MÁ, Boguná M. Clustering in complex networks. II. Percolation properties. Physical Review E. 2006;74(5):056115 10.1103/PhysRevE.74.056115 17279976

[41] Granovetter MS. The strength of weak ties. American Journal of Sociology. 1973;p. 1360–1380. 10.1086/225469

[42] Montgomery JD. Job search and network composition: Implications of the strength-of-weak-ties hypothesis. American Sociological Review. 1992;p. 586–596. 10.2307/2095914

[43] Onnela JP, Saramäki J, Hyvönen J, Szabó G, Lazer D, Kaski K, et al Structure and tie strengths in mobile communication networks. Proceedings of the National Academy of Sciences. 2007;104(18):7332–7336. 10.1073/pnas.0610245104 PMC1863470 17456605

[44] Gallos LK, Makse HA, Sigman M. A small world of weak ties provides optimal global integration of self-similar modules in functional brain networks. Proceedings of the National Academy of Sciences. 2012;109(8):2825–2830. 10.1073/pnas.1106612109 PMC3286928 22308319

[45] Masuda N. Immunization of networks with community structure. New Journal of Physics. 2009;11(12):123018 10.1088/1367-2630/11/12/123018

[46] Newman ME. Modularity and community structure in networks. Proceedings of the National Academy of Sciences. 2006;103(23):8577–8582. 10.1073/pnas.0601602103 PMC1482622 16723398

