# Supplementary material for: Finding Influential Spreaders from Human Activity beyond Network Location
Source: PLoS One. 2015 Aug 31;10(8):e0136831. doi: 10.1371/journal.pone.0136831 (PMC4554996; doi:10.1371/journal.pone.0136831)
Supplement: S1 File — Table A, Multilinear regression for the QXF networks with Eq (1). Table B, Multilinear regression for the QXG networks with Eq (1). Table C, Multilinear regression for the POK networks with Eq (1). Table D, Multilinear regression for the LJ networks with Eq (1). Table E, Multilinear regression for the PRO networks with Eq (1). Table F, Multilinear regression for the QXF networks with Eqs (2) and (5). Table G, Multilinear regression for the QXG networks with Eqs (2) and (5). Table H, Multilinear regression for the POK networks with Eqs (2) and (5). Table I, Multilinear regression for the LJ networks with Eqs (2) and (5). Table J, Multilinear regression for the PRO networks with Eqs (2) and (5). Text A, k-shell index. Text B, Identifying structural hole in the link community. (PDF) [file pone.0136831.s001.pdf]

Supporting information S1 File

Finding influential spreaders from human activity beyond network location

Byungjoon Min[1,*], Fredrik Liljeros[2,3], Hernán A. Makse[1]

1 Levich Institute and Physics Department, City College of New York, New York, NY, US

2 Department of Sociology, Stockholm University, Stockholm, Sweden

3 Institute for Futures Study, Stockholm, Sweden

*** E-mail: min.byungjoon@gmail.com**

Table A. Multilinear regression for the QXF network with Eq. (1).

$M_i = c_0 + c_1 a_i^{exc} + c_2 a_i^{bal} + c_3 a_i^{ca} + c_4 a_i^{sh} + c_5 k_i + c_6 k_i^{sh} + c_7 k_i^{2sum} + c_8 k_i^{sum} + \epsilon$.

intercept	k_{sh}	k_{2sum}	k_{sum}	k	exchange	balance	col. act.	str. hole	R^2
−0.953***	0.118***	6.82e-6***	5.63e-5***	4.41e-3***	−0.353***	0.330***	0.153*	−0.379***	0.950
[0.0199]	[1.41e-3]	[1.22e-7]	[2.76e-6]	[1.65e-4]	[0.0253]	[0.0258]	[0.0390]	[0.0781]	-
−0.0859***	-	1.49e-5***	−2.51e-5***	6.01e-3***	−0.374***	0.766***	0.0890^{NS}	−1.02***	0.923
[0.0211]	-	[9.22e-8]	[3.19e-6]	[2.02e-4]	[0.0313]	[0.0312]	[0.0482]	[0.0962]	-
−1.20***	0.181***	-	1.64e-4***	2.40e-3***	−0.367***	0.103***	0.359***	−0.298***	0.938
[0.0216]	[9.64e-4]	-	[2.21e-6]	[1.79e-4]	[0.0281]	[0.0283]	[0.0432]	[0.0869]	-
−0.983***	0.108***	8.54e-6***	-	6.88e-3***	−0.338***	0.388***	0.198***	−0.414***	0.948
[0.0202]	[1.34e-3]	[8.89e-8]	-	[1.14e-4]	[0.0256]	[0.0260]	[0.0395]	[0.0793]	-
−0.868***	0.123***	6.11e-6***	1.11e-4***	-	−0.402***	0.350***	−0.0573^{NS}	−0.330***	0.947
[0.0202]	[1.44e-3]	[1.22e-7]	[1.92e-6]	-	[0.0259]	[0.0264]	[0.0392]	[0.0801]	-
−1.03***	0.118***	6.84e-6***	5.52e-5***	4.58e-3***	-	0.264***	0.197***	−0.426***	0.949
[0.0194]	[1.42e-3]	[1.22e-7]	[2.78e-6]	[1.65e-4]	-	[0.0255]	[0.0391]	[0.0786]	-
−0.961***	0.122***	6.58e-6***	6.02e-5***	4.47e-3***	−0.294***	-	0.235***	−0.511***	0.949
[0.0200]	[1.39e-3]	[1.21e-7]	[2.76e-6]	[1.66e-4]	[0.0250]	-	[0.0387]	[0.0779]	-
−0.907***	0.118***	6.87e-6***	5.70e-5***	4.28e-3***	−0.361***	0.347***	-	−0.313***	0.950
[0.0161]	[1.41e-3]	[1.21e-7]	[2.76e-6]	[1.61e-4]	[0.0252]	[0.0254]	-	[0.0763]	-
−0.963***	0.119***	6.81e-6***	5.66e-5***	4.39e-3***	−0.358***	0.347***	−0.112**	-	0.950
[0.0199]	[1.41e-3]	[1.22e-7]	[2.76e-6]	[1.65e-4]	[0.0253]	[0.0256]	[0.0381]	-	-
−2.05***	0.281***	-	-	-	-	-	-	-	0.823
[0.0247]	[0.00114]	-	-	-	-	-	-	-	-
−0.121***	-	1.64e-5***	-	-	-	-	-	-	0.908
[0.0126]	-	[4.54-8]	-	-	-	-	-	-	-
1.49***	-	-	3.54e-4***	-	-	-	-	-	0.757
[0.0160]	-	-	[1.75-6]	-	-	-	-	-	-
1.92***	-	-	-	0.0280***	-	-	-	-	0.580
[0.0202]	-	-	-	[0.000208]	-	-	-	-	

Coefficients [standard errors] for the linear models. Here, $***$ denotes $p < 0.001$, $**$ denotes $p < 0.01$, $*$ denotes $p < 0.05$, and NS denotes not significant with significant level of p-value 0.05.

Table B. Multilinear regression for the QXG network with Eq. (1).
$M_i = c_0 + c_1 a_i^{exc} + c_2 a_i^{bal} + c_3 a_i^{ca} + c_4 a_i^{sh} + c_5 k_i + c_6 k_i^{sh} + c_7 k_i^{2sum} + c_8 k_i^{sum} + \epsilon$.

intercept	k_{sh}	k_{2sum}	k_{sum}	k	exchange	balance	coll. act.	str. hole	R^2
−0.831***	0.238***	5.97e-5***	−5.16e-4***	1.20e-2***	−4.02e-3^{NS}	0.400***	0.157***	−0.295***	0.966
[0.0206]	[2.67e-3]	[7.71e-7]	[1.88e-5]	[3.86e-4]	[0.0210]	[0.0276]	[0.0435]	[0.0580]	
0.268***		1.09e-4***	−1.38e-3***	1.79e-2***	0.248***	0.951***	0.339***	−0.852***	0.934
[0.0228]		[7.45e-7]	[2.22e-5]	[5.26e-4]	[0.0288]	[0.0372]	[0.0601]	[0.0797]	
−1.31***	0.386***		7.48e-4***	5.66e-3***	−0.179***	0.0961**	0.436***	−2.27**	0.942
[0.0254]	[2.42e-3]		[1.20e-5]	[4.90e-4]	[0.0271]	[0.0354]	[0.0563]	[0.0753]	
−0.902***	0.277***	4.12e-5***		5.67e-3***	−0.0801***	0.313***	0.149**	−2.63***	0.963
[0.0213]	[2.38e-3]	[3.95e-7]		[3.24e-4]	[0.0217]	[0.0285]	[0.0453]	[0.0604]	
−0.764***	0.253***	5.46e-5***	−1.72e-4***		−0.104***	0.392***	−0.0513^{NS}	−0.270***	0.962
[0.0215]	[2.77e-3]	[7.94e-7]	[1.59e-5]		[0.0219]	[0.0290]	[0.0453]	[0.0611]	
−0.832***	0.238***	5.97e-5***	−5.17e-4***	1.20e-2***		0.400***	0.155***	−0.295***	0.966
[0.0205]	[2.65e-3]	[7.67e-6]	[1.86e-5]	[3.81e-4]		[0.0274]	[0.0423]	[0.0579]	
−0.864***	0.247***	5.81e-5***	−4.85e-4***	1.19e-2***	0.315^{NS}		0.285***	−0.449***	0.965
[0.0207]	[2.63e-3]	[7.72e-7]	[1.89e-5]	[3.91e-4]	[0.0211]		[0.0431]	[0.0577]	
−0.796***	0.239***	5.99e-5***	−5.16e-4***	1.17e-2***	0.0137^{NS}	0.420***		−2.20***	0.966
[0.0182]	[2.67e-3]	[7.69e-7]	[1.88e-5]	[3.82e-4]	[0.0204]	[0.0270]		[0.0542]	
−0.833***	0.240***	5.96e-5***	−5.14e-4***	1.19e-2***	−0.00865^{NS}	0.426***	0.0781^{NS}		0.966
[0.0206]	[2.66e-3]	[7.72e-7]	[1.88e-5]	[3.86e-4]	[0.0210]	[0.0271]	[0.0407]		
−2.22***	0.600***								0.815
[0.0351]	[0.00306]								
0.918***		7.08e-5***							0.896
[0.0160]		[2.58-7]							
1.79***			1.58e-3***						0.750
[0.0224]			[9.76-6]						
2.09***				0.0628***					0.624
[0.0269]				[0.000522]					

Coefficients [standard errors] for the linear models. Every row corresponds to a different model. Here, $***$ denotes $p < 0.001$, $**$ denotes $p < 0.01$, $*$ denotes $p < 0.05$, and NS denotes not significant.

Table C. Multilinear regression for the POK network with Eq. (1).
$M_i = c_0 + c_1 a_i^{exc} + c_2 a_i^{bal} + c_3 a_i^{ca} + c_4 a_i^{sh} + c_5 k_i + c_6 k_i^{sh} + c_7 k_i^{2sum} + c_8 k_i^{sum}$.

intercept	k_{sh}	k_{2sum}	k_{sum}	k	exchange	balance	coll. act.	str. hole	R^2
−1.19***	0.457***	2.62e-5***	−1.99e-4***	7.88e-3***	−0.0827*	−0.0819*	0.813***	−0.594***	0.907
[0.0159]	[2.56e-3]	[1.03e-6]	[1.14e-5]	[1.71e-4]	[0.0205]	[0.0401]	[0.0360]	[0.0456]	
−0.679***		1.63e-4***	−1.50e-3***	2.33e-2***	0.165***	0.651***	1.05***	−1.72***	0.727
[0.0267]		[1.19e-6]	[1.51e-5]	[2.52e-4]	[0.0350]	[0.0682]	[0.0614]	[0.0773]	
−1.16***	0.505***		8.02e-5***	5.51e-3***	−0.0873***	−0.106**	0.887***	−0.509***	0.903
[0.0161]	[1.75e-3]		[3.11e-6]	[1.45e-4]	[0.0209]	[0.0408]	[0.0365]	[0.0464]	
−1.16***	0.485***	8.83e-6***		6.12e-3***	−0.0881***	−0.111**	0.853***	−5.70***	0.905
[0.0159]	[1.99e-3]	[2.78e-7]		[1.39e-4]	[0.0207]	[0.0404]	[0.0362]	[0.0460]	
−1.16***	0.516***	4.95e-8^{NS}	1.12e-4***		−0.107***	−0.110**	0.777***	−0.609***	0.895
[0.0168]	[2.34e-3]	[9.17e-7]	[9.81e-6]		[0.0218]	[0.0426]	[0.0382]	[0.0485]	
−1.21***	0.456***	2.62e-5***	−2.00e-4***	7.89e-3***		−0.0952*	0.807***	−0.599***	0.906
[0.0151]	[2.55e-3]	[1.03e-6]	[1.14e-5]	[1.71e-4]		[0.0400]	[0.0360]	[0.0456]	
−1.19***	0.456***	2.62e-5***	−2.00e-4***	7.88e-3***	−0.0861***		0.809***	−0.586***	0.907
[0.0159]	[2.54e-3]	[1.03e-6]	[1.14e-5]	[1.71e-4]	[0.0204]		[0.0359]	[0.0455]	
0.963***	0.459***	2.81e-5***	−2.16e-4***	7.79e-3***	−0.0620**	−0.0322^{NS}		−0.229***	0.904
[0.0126]	[2.59e-3]	[1.04e-6]	[1.16e-5]	[1.73e-4]	[0.0208]	[0.0406]		[0.0433]	
−1.17***	0.461***	2.52e-5***	−1.95e-4***	7.89e-3***	−0.0906***	0.0399^{NS}	0.648***		0.906
[0.0159]	[2.54e-3]	[1.03e-6]	[1.15e-5]	[1.72e-4]	[0.0206]	[0.0402]	[0.0338]		
−1.00***	0.555***								0.883
[0.0115]	[0.00157]								
0.638***		5.81e-5***							0.481
[0.0196]		[4.69-7]							
1.42***			5.91e-4***						0.326
[0.0189]			[6.59-6]						
2.14***				0.0230***					0.180
[0.0173]				[0.000380]					

Coefficients [standard errors] for the linear models. Every row corresponds to a different model. Here, $***$ denotes $p < 0.001$, $**$ denotes $p < 0.01$, $*$ denotes $p < 0.05$, and NS denotes not significant.

Table D. Multilinear regression for the LJ network with Eq. (1).
$M_i = c_0 + c_1 a_i^{exc} + c_2 a_i^{bal} + c_3 a_i^{ca} + c_4 a_i^{sh} + c_5 k_i + c_6 k_i^{sh} + c_7 k_i^{2sum} + c_8 k_i^{sum}$.

intercept	k_{sh}	k_{2sum}	k_{sum}	k	exchange	balance	coll. act.	str. hole	R^2
0.0698***	0.0887***	7.79e-6***	−5.76e-5***	1.61e-3***	0.386*	0.307***	−2.39***	−0.129***	0.682
[0.0138]	[5.41e-4]	[4.34e-8]	[5.78e-7]	[3.93e-5]	[0.0699]	[0.0366]	[0.0345]	[0.0373]	
0.396***		7.99e-6***	−4.51e-5***	2.48e-3***	0.912***	2.99***	−0.277***	−2.11***	0.551
[0.0162]		[5.16e-8]	[6.81e-7]	[4.63e-5]	[0.0830]	[0.0389]	[0.0411]	[0.0439]	
0.782***	0.0913***		3.43e-5***	−1.76e-5^{NS}	0.301***	0.721***	−0.0732^{NS}	0.343***	0.525
[0.0161]	[6.61e-4]		[3.27e-7]	[4.67e-5]	[0.0855]	[0.0446]	[0.0422]	[0.0442]	
0.431***	0.0816***	3.95e-6***		3.99e-4***	0.292***	0.552***	−0.361***	−0.981***	0.634
[0.0143]	[5.76e-4]	[2.16e-8]		[4.01e-5]	[0.0750]	[0.0392]	[0.0371]	[0.0399]	
0.128***	0.0916***	7.38e-6***	−5.03e-5***		0.344***	0.332***	−0.342***	−1.30***	0.674
[0.0139]	[5.43e-4]	[4.28e-8]	[5.56e-7]		[0.0708]	[0.0370]	[0.0349]	[0.0377]	
0.0768***	0.0888***	7.79e-6***	−5.75e-5***	1.61e-3***		0.331***	−0.224***	−1.28***	0.681
[0.0137]	[5.40e-4]	[4.34e-8]	[5.78e-7]	[3.93e-5]		[0.0363]	[0.0345]	[0.0373]	
0.0558***	0.0907***	7.81e-6***	−5.79e-5***	1.62e-3***	0.455***		−0.152***	−1.33**	0.682
[0.0137]	[4.84e-4]	[4.34e-8]	[5.77e-7]	[3.93e-5]	[0.0694]		[0.0329]	[0.0370]	
0.00122^{NS}	0.0887***	7.78e-6***	−5.77e-5***	1.63e-4***	0.400***	0.231***		−0.136*	0.682
[0.00956]	[5.41e-4]	[4.34e-8]	[5.77e-7]	[3.92e-5]	[0.0699]	[0.0349]		[0.0357]	
−0.103***	0.0912***	7.43e-6***	−5.59e-5***	1.62e-3***	0.331***	0.475***	−0.584***		0.676
[0.0139]	[5.41e-4]	[4.25e-8]	[5.81e-7]	[3.96e-5]	[0.0705]	[0.0366]	[0.0334]		
1.17***	0.123***								0.420
[0.00986]	[0.000568]								
0.713***		4.99e-6***							0.436
[0.0109]		[2.22e-8]							
1.40***			5.57e-5***						0.310
[0.0105]			[3.26e-7]						
2.27***				0.00363***					0.0501
[0.0106]				[6.20e-5]					

Coefficients [standard errors] for the linear models. Here, $***$ denotes $p < 0.001$, $**$ denotes $p < 0.01$, $*$ denotes $p < 0.05$, and NS denotes not significant with significant level of p-value 0.05.

Table E. Multilinear regression for the PRO network with Eq. (1).
$M_i = c_0 + c_1 a_i^{exc} + c_2 a_i^{bal} + c_3 a_i^{ca} + c_4 a_i^{sh} + c_5 k_i + c_6 k_i^{sh} + c_7 k_i^{2sum} + c_8 k_i^{sum}$.

intercept	k_{sh}	k_{2sum}	k_{sum}	k	coll. act.	str. hole	R^2
-1.12^{***}	0.610^{***}	$3.76e\text{-}4^{***}$	$1.24e\text{-}4^{***}$	$1.25e\text{-}2^{***}$	1.13^{***}	-0.630^{***}	0.945
[0.0204]	[5.36e-3]	[5.09e-6]	[3.58e-5]	[7.59e-4]	[0.0361]	[0.0556]	
0.371^{***}		$6.35e\text{-}4^{***}$	$9.69e\text{-}4^{***}$	$2.74e\text{-}2^{***}$	0.604^{***}	0.626^{***}	0.866
[0.0242]		[7.08e-6]	[5.45e-5]	[1.16e-3]	[0.0557]	[0.0846]	
-1.58^{***}	0.787^{***}		$1.29e\text{-}3^{***}$	$2.75e\text{-}2^{***}$	1.69^{***}	0.107^{NS}	0.912
[0.0246]	[6.06e-3]		[4.06e-5]	[9.24e-4]	[0.0446]	[0.0691]	
-1.15^{***}	0.614^{***}	$3.84e\text{-}4^{***}$		$1.32e\text{-}2^{***}$	1.164^{***}	-0.659^{***}	0.945
[0.0193]	[5.25e-3]	[4.57e-6]		[7.40e-4]	[0.0347]	[0.0550]	
-1.05^{***}	0.625^{***}	$3.99e\text{-}4^{***}$	$2.58e\text{-}4^{***}$		0.899^{***}	-0.789^{***}	0.943
[0.0203]	[5.36e-3]	[4.98e-6]	[3.54e-5]		[0.0338]	[0.0555]	
-0.672^{***}	0.589^{***}	$4.09e\text{-}4^{***}$	$4.38e\text{-}4^{**}$	$3.45e\text{-}3^{***}$		-0.316^{***}	0.939
[0.0152]	[5.59e-3]	[5.24e-6]	[3.62e-5]	[7.37e-4]		[0.0575]	
-1.10^{***}	0.598^{***}	$3.66e\text{-}4^{***}$	$1.85e\text{-}4^{***}$	$1.40e\text{-}2^{***}$	1.05^{***}		0.944
[0.0204]	[5.29e-3]	[5.04e-6]	[3.56e-5]	[7.53e-4]	[0.0358]		
-1.44^{***}	1.07^{***}						0.859
[0.0199]	[0.00456]						
0.621^{***}		$8.40e\text{-}4^{***}$					0.848
[0.0138]		[3.71e-6]					
1.25^{***}			$6.12e\text{-}3^{***}$				0.669
[0.0186]			[4.50e-5]				
1.52^{***}				0.134^{***}			0.452
[0.0238]				[0.00154]			

Coefficients [standard errors] for the linear models. Here, $***$ denotes $p < 0.001$, $**$ denotes $p < 0.01$, $*$ denotes $p < 0.05$, and NS denotes not significant with significant level of p-value 0.05.

Table F. Multilinear regression for the QXF network with Eqs. (2) and (5).
$M_i = c_0 + c_1 a_i^{exc} + c_2 a_i^{bal} + c_3 a_i^{ca} + c_4 a_i^{sh} + c_5 k_i$.

intercept	k	exchange	balance	coll. act.	str. hole	R^2
0.889***	0.0268***	−0.416***	1.11***	2.89***	−3.79***	0.625
[0.0432]	[0.000205]	[0.0690]	[0.0688]	[0.103]	[0.211]	
0.808***	0.0270***	-	1.04***	2.94***	−3.84***	0.624
[0.0410]	[0.000204]		[0.0677]	[0.102]	[0.211]	
0.954***	0.0276***	−0.209**	-	3.20***	−4.34***	0.617
[0.0433]	[0.000201]	[0.0685]		[0.102]	[0.210]	
1.86***	0.0265***	−0.579***	1.48***	-	−2.63***	0.602
[0.0269]	[0.000211]	[0.0708]	[0.0696]		[0.213]	
0.868***	0.0272***	−0.473***	1.32***	2.53***	-	0.615
[0.0436]	[0.000207]	[0.0675]	[0.0687]	[0.102]		
2.40***	-	−1.39***	3.26***	2.06***	−6.12***	0.133
[0.0631]		[0.104]	[0.102]	[0.156]	[0.319]	
2.15***	-	-	3.04***	2.22***	−6.36***	0.121
[0.0607]			[0.101]	[0.156]	[0.321]	
2.74***	-	−0.843***	-	2.95***	−8.07***	0.0650
[0.0647]		[0.107]		[0.159]	[0.326]	
3.07***	-	−1.50***	3.50***	-	−5.28***	0.121
[0.0373]		[0.105]	[0.101]		[0.314]	
2.39***	-	−1.51***	3.63***	1.46***	-	0.109
[0.0640]		[0.105]	[0.101]	[0.155]		

Coefficients [standard errors] for the linear models. Here, $***$ denotes $p < 0.001$, $**$ denotes $p < 0.01$, $*$ denotes $p < 0.05$, and NS denotes not significant with significant level of p-value 0.05.

Table G. Multilinear regression for the QXG network with Eqs. (2) and (5).
$M_i = c_0 + c_1 a_i^{exc} + c_2 a_i^{bal} + c_3 a_i^{ca} + c_4 a_i^{sh} + c_5 k_i$.

intercept	k	exchange	balance	coll. act.	str. hole	R^2
0.63***	0.0600***	0.517**	1.25***	3.24***	−2.41***	0.684
[0.0482]	[0.000490]	[0.0628]	[0.0815]	[0.127]	[0.174]	
0.71***	0.0597***		1.35***	3.53***	−2.38***	0.682
[0.0474]	[0.000491]		[0.0808]	[0.122]	[0.174]	
0.657***	0.0611***	0.668***		3.71***	−3.00***	0.675
[0.0488]	[0.000491]	[0.0629]		[0.125]	[0.172]	
1.46***	0.0608***	0.960***	1.75***		−0.893***	0.660
[0.0369]	[0.000507]	[0.0626]	[0.0820]		[0.170]	
0.681***	0.0608***	0.496***	1.50***	2.64***		0.677
[0.0486]	[0.000493]	[0.0635]	[0.0804]	[0.121]		
2.10***		−0.102***	2.72***	4.23***	−4.81***	0.141
[0.0769]		[0.103]	[0.133]	[0.209]	[0.285]	
2.08***			2.70***	4.17***	−4.82***	0.141
[0.0756]			[0.131]	[0.201]	[0.285]	
2.21***		0.208***		5.31***	−6.22***	0.100
[0.0785]		[0.104]		[0.207]	[0.283]	
3.21***		0.467***	3.40***		−2.86***	0.101
[0.0552]		[0.102]	[0.132]		[0.275]	
2.23***		−0.161^{NS}	3.26***	3.04***		0.113
[0.0778]		[0.105]	[0.131]	[0.200]		

Coefficients [standard errors] for the linear models. Here, $***$ denotes $p < 0.001$, $**$ denotes $p < 0.01$, $*$ denotes $p < 0.05$, and NS denotes not significant with significant level of p-value 0.05.

Table H. Multilinear regression for the POK network with Eqs. (2) and (5).
$M_i = c_0 + c_1 a_i^{exc} + c_2 a_i^{bal} + c_3 a_i^{ca} + c_4 a_i^{sh} + c_5 k_i$.

intercept	k	exchange	balance	coll. act.	str. hole	R^2
0.669***	0.0222***	0.691***	2.29***	3.15***	−1.34***	0.264
[0.0414]	[0.000362]	[0.0571]	[0.110]	[0.0987]	[0.126]	
0.859***	0.0223***		2.44***	3.23***	−1.31***	0.257
[0.0385]	[0.000364]		[0.110]	[0.0989]	[0.126]	
0.738***	0.0228***	0.817***		3.34***	−1.57***	0.245
[0.0418]	[0.000365]	[0.0575]		[0.0995]	[0.127]	
1.67***	0.0221***	0.814***	2.62***		0.140***	0.219
[0.0278]	[0.000373]	[0.0587]	[0.113]		[0.121]	
0.713***	0.0223***	0.675***	2.39***	2.77***		0.259
[0.0413]	[0.000363]	[0.0573]	[0.110]	[0.0920]		
0.921***		0.760***	2.85***	3.13***	−1.67***	0.0977
[0.0456]		[0.0632]	[0.122]	[0.109]	[0.139]	
1.13***			3.01***	3.21***	−1.63***	0.0898
[0.0423]			[0.121]	[0.109]	[0.140]	
1.02***		0.920***		3.36***	−1.96***	0.0680
[0.0461]		[0.0639]		[0.111]	[0.141]	
1.91***		0.882***	3.17***		-0.19^{NS}	0.0532
[0.0303]		[0.0646]	[0.124]		[0.133]	
0.978***		0.741***	2.98***	2.64***		0.0899
[0.0455]		[0.0635]	[0.122]	[0.102]		

Coefficients [standard errors] for the linear models. Here, $***$ denotes $p < 0.001$, $**$ denotes $p < 0.01$, $*$ denotes $p < 0.05$, and NS denotes not significant with significant level of p-value 0.05.

Table I. Multilinear regression for the LJ network with Eqs. (2) and (5).
$M_i = c_0 + c_1 a_i^{exc} + c_2 a_i^{bal} + c_3 a_i^{ca} + c_4 a_i^{sh} + c_5 k_i$.

intercept	k	exchange	balance	coll. act.	str. hole	R^2
1.24***	0.00305***	1.30**	4.47***	0.804***	1.46***	0.195
[0.0207]	[5.76e-5]	[0.111]	[0.0513]	[0.0545]	[0.0549]	
1.26***	0.00305***		4.56***	0.787***	1.48***	0.194
[0.0206]	[5.76e-5]		[0.0507]	[0.0545]	[0.0549]	
1.18***	0.00366***	2.88***		2.52***	0.638***	0.103
[0.0219]	[6.04e-5]	[0.116]		[0.0537]	[0.0571]	
1.48***	0.00301***	1.26***	4.74***		1.75***	0.193
[0.0129]	[5.76e-5]	[0.111]	[0.0479]		[0.0512]	
1.23***	0.00304***	1.38***	4.23***	1.33***		0.186
[0.0208]	[5.79e-5]	[0.112]	[0.0508]	[0.0510]		
1.32***		1.32***	4.80***	0.671***	1.43***	0.160
[0.0211]		[0.113]	[0.0520]	[0.0556]	[0.0560]	
1.35***			4.89***	0.6534$***$	1.45***	0.159
[0.0210]			[0.0514]	[0.0556]	[0.0561]	
1.27***		3.03***		2.51***	0.531***	0.0505
[0.0224]		[0.119]		[0.0551]	[0.0587]	
1.52***		1.28**	5.02***		1.68***	0.158
[0.0132]		[0.113]	[0.0486]		[0.0523]	
1.31***		1.39**	4.56***	1.19***		0.152
[0.0212]		[0.114]	[0.0515]	[0.0520]		

Coefficients [standard errors] for the linear models. Here, $***$ denotes $p < 0.001$, $**$ denotes $p < 0.01$, $*$ denotes $p < 0.05$, and NS denotes not significant with significant level of p-value 0.05.

Table J. Multilinear regression for the PRO network with Eqs. (2) and (5).
$M_i = c_0 + c_1 a_i^{exc} + c_2 a_i^{bal} + c_3 a_i^{ca} + c_4 a_i^{sh} + c_5 k_i$.

intercept	k	coll. act.	str. hole	R^2
-0.405^{***}	0.137^{***}	3.83^{***}	3.67^{***}	0.625
[0.0382]	[0.00128]	[0.0810]	[0.134]	
-0.412^{***}	0.139^{***}		4.53^{***}	0.594
[0.0398]	[0.00133]		[0.0800]	
1.07^{***}	0.131^{***}	5.65^{***}		0.533
[0.0246]	[0.00142]	[0.142]		
0.891^{***}		3.10^{***}	4.50^{***}	0.154
[0.0545]		[0.121]	[0.201]	
2.05^{***}			6.07^{***}	0.093
[0.0309]			[0.198]	
0.906^{***}		3.93^{***}		0.108
[0.0559]		[0.118]		

Coefficients [standard errors] for the linear models. Here, $***$ denotes $p < 0.001$, $**$ denotes $p < 0.01$, $*$ denotes $p < 0.05$, and NS denotes not significant with significant level of p-value 0.05.

Text A. k-shell index

In order to assign k-shell index, we remove all nodes with degree less than k until no nodes less than k remains. Then the maximum subgraph whose remained degree is larger than k is called k-core. k-shell is the set of all nodes belonging to the k-core but not to the $(k+1)$-core. As a result, each node is assigned with a unique k-shell index.

Text B. Identifying structural hole in the link community

In order to identify the intercommunity links, called the structural hole, we use the link community detection method proposed in [1]. We adapt the method for local version only using local information of networks. When a new link e_{ik} is added, likewise the original link community algorithm [1], we define the similarity $S(e_{ik}, e_{jk})$ between two links e_{ik} and each of existed links e_{jk} by following,

$$S(e_{ik}, e_{jk}) = \frac{|n_+(i) \cap n_+(j)|}{|n_+(i) \cup n_+(j)|}, \tag{1}$$

where $n_+(i)$ is the set of neighbors of node i. If the similarity is less than a certain threshold meaning that two neighbors have only few fraction of common friends, we judge the newly added link as a structural hole.

References

1. Ahn YY, Bagrow JP, Lehmann S, Link communities reveal multiscale complexity in networks, Nature. 2010;466(7307):761-764.
